# Gut microbiota and inflammatory factor characteristics in major depressive disorder patients with anorexia

**DOI:** 10.1186/s12888-024-05778-0

**Published:** 2024-05-02

**Authors:** Fengtao Guo, Lin Jing, Yunfan Xu, Kun Zhang, Ying Li, Ning Sun, Penghong Liu, Huanhu Zhang

**Affiliations:** 1 Department of Psychiatry, First Hospital of Shanxi Medical University, Taiyuan, 030001 China; 2 Shanxi Medical University, Taiyuan, 030001 China; 3 Yanhu District Branch, The First Hospital of Shanxi Medical University, Yuncheng, 044000 China; 4 Shanxi University of Chinese Medicine, Jinzhong, 030619 China

**Keywords:** Major depressive disorder, Anorexia, Gut microbiota, Inflammatory factors

## Abstract

**Background:**

This study aimed to explore the gut microbiota and inflammatory factor characteristics in major depressive disorder (MDD) patients with anorexia and to analyze the correlation between gut microbiota and inflammatory factors, anorexia, and HAMD scores.

**Methods:**

46 MDD patients and 46 healthy controls (HC) were included in the study. The 46 MDD patients were divided into two groups according to whether they had anorexia:20 MDD without anorexia (MDA0 group) and 26 MDD with anorexia (MDA1 group). We used the Hamilton Depression Scale-24 (HAMD-24) to evaluate the depression status of all participants and 16 S ribosomal RNA (16 S rRNA)sequencing to evaluate the composition of the gut microbiota. Inflammatory factors in peripheral blood such as C-reactive protein (CRP) were detected using enzyme-linked immunosorbent assay (ELISA). Spearman’s correlation analysis was used to evaluate the correlation between gut microbiota and inflammatory factors, HAMD scores, and anorexia.

**Results:**

1). CRP was significantly higher in the MDA0, MDA1, than HC. 2). An analysis of α-diversity shows: the Simpson and Pielou indices of the HC group are higher than the MDA1 group (*P* < 0.05). 3). The β-diversity analysis shows differences in the composition of microbial communities between the MDA0, MDA1, and HC group. 4). A correlation analysis showed that Blautia positively correlated with anorexia, HAMD scores, and CRP level, whereas Faecalibacterium, Bacteroides, Roseburia, and Parabacteroides negatively correlated with anorexia, HAMD scores, and CRP level. 5). The receiver operating characteristic (ROC) curve was drawn using the differential bacterial genera between MDD patients with or without anorexia as biomarkers to identify whether MDD patients were accompanied with anorexia, and its area under curve (AUC) was 0.85. The ROC curve was drawn using the differential bacterial genera between MDD patients with anorexia and healthy controls as biomarkers to diagnose MDD patients with anorexia, with its AUC was 0.97.

**Conclusion:**

This study suggested that MDD patients with anorexia had a distinct gut microbiota compared to healthy individuals, with higher level of CRP. Blautia was more abundant in MDD patients with anorexia and positively correlated with CRP, HAMD scores, and anorexia. The gut microbiota might have influenced MDD and anorexia through the inflammatory factor CRP.

## Background

Major depressive disorder (MDD) is a common mental disorder characterized by persistently low mood, with a relatively high disability rate and high risk of suicide [1, 2]. Approximately 322 million people worldwide suffer from depression, accounting for 4.4% of the global population [3]. It is estimated to become the leading disease-related burden worldwide by 2030 [4]. Therefore, depression seriously affects people’s quality of life and physical health and places a heavy burden on individuals and society.

MDD is often accompanied with anorexia [5]. Similarly, many anorexia patients will have MDD [6, 7]. Being one characteristic of MDD, anorexia is closely related to MDD [8]. Reports suggest that approximately half of MDD patients experience a decrease in appetite [9], while more than 40% of anorexia patients also have comorbid MDD [7, 10]. Studies have shown that MDD is the most common comorbidity in anorexia patients [7]. Furthermore, research [11] indicates that MDD is related to the clinical severity of anorexia, and ameliorating depression symptoms can relieve anorexia symptoms and vice versa [12]. However, some studies have shown that depressive symptoms do not influence the short-term treatment effects in patients with anorexia nervosa [13]. In summary, depressive and anorexic symptoms are closely related, suggesting that depressive symptoms in MDD patients may share a common pathogenic mechanism with anorexic symptoms. At present, the pathogenesis of depression has not been fully clarified, however, related studies have shown that inflammation plays a very important role in the occurrence and development of depression, and targeting inflammation may become a new direction for the treatment of depression [14].

With the continuous deepening of research on gut microbiota, previous studies have found that gut microbiota can affect the brain by regulating the immune system, producing neurotransmitters, and activating the nervous system, and that the brain-gut axis plays an important role in central nervous system (CNS) diseases [15]. Both clinical and animal studies have shown changes in the gut microbiota in MDD, and altered microbiota may be involved in the pathogenesis of MDD [16, 17]. Although there is no unified conclusion, many studies have shown that the occurrence of MDD is accompanied with changes in inflammatory factors, and with a higher abundance of pro-inflammatory bacteria and lower abundance of anti-inflammatory bacteria [16, 18]. After transplanting the feces of MDD patients into germ-free mice, the mice exhibited depressive behaviors [17]. Similarly, transplanting fecal bacteria from healthy people into depressed mice can ameliorate depressive symptoms [19], which strongly suggests that gut microbiota may be responsible for MDD. Zhang [20]found that, after 14 days of exposure to psychological stress, mice displayed depressive-like behaviors. Compared to non-stressed mice, the abundance of Bacteroides, Alistipes, and Lactobacillus in the gut microbiota of stressed mice decreased, while Parasutterella increased. Through review analyses, scholars have concluded that Coprococcus, Eggerthella, Subdoligranulum, Hungatella, Sellimonas, Sutterella, and Eubacterium are related to depression at the genus level [21]. Individuals with more severe depressive symptoms have higher Sellimonas, Eggerthella, and Hungatella [22].

In summary, gut microbiota is involved in the occurrence and development of depression, which is often accompanied by changes in inflammatory factors. MDD and anorexia often occur simultaneously, therefore, MDD accompanied with anorexia may become a subtype of MDD with unique biological characteristics. Previous research has mainly focused on the relationship between gut microbiota and depression or anorexia, and few studies have been conducted on depression patients with anorexia. Therefore, we studied the characteristics of the gut microbiota and inflammatory factors in MDD patients with anorexia and analyzed their correlations to provide new strategies for the treatment of MDD patients with anorexia.

## Methods

### Participants

After excluding cases with incomplete data, 46 first-episode, untreated MDD patients were recruited from the in- and outpatient units of the First Hospital of Shanxi Medical University Department of Psychiatry from August 2019 to September 2022, as well as 46 healthy controls matched by sex, age, years of education, and body mass index(BMI). All MDD patients were diagnosed using the Diagnostic and Statistical Manual of Mental Disorders, Fourth Edition (DSM-IV). The Hamilton Depression Scale-24 (HAMD-24) was used to measure depression severity, and all MDD patients had scores of > 20. 46 eligible MDD patients were divided into two groups according to whether they had anorexia based on the item 12 of HAMD-24: 20 MDD without anorexia (MDA0 group) and 26 MDD with anorexia (MDA1 group). 46 cases of healthy controls(HC) with normal appetite and without any diseases were recruited from the Physical Examination Center of the First Hospital of Shanxi Medical University. Subjects were excluded under the following conditions:1. Suffering from other mental diseases, such as schizophrenia and bipolar disorder; 2. Patients with other physical diseases such as hypertension, diabetes, endocrine diseases, and gastrointestinal diseases; 3. Antibiotic or probiotic preparations one month before enrollment; 4. Pregnancy or lactation.

All human participants involved in this study were approved by the Medical Ethics Committee of the First Hospital of Shanxi Medical University. All participants provided written informed consent after receiving a detailed description of the research objectives and procedures.

### Collection of fecal and blood samples

After the assessment of all participants using HAMD-24, fecal samples were collected and immediately stored frozen at − 80 °C, and the gut microbiota was detected using the 16 S ribosomal RNA (16 S rRNA) gene sequencing method. We used sterile techniques to extract 10 ml of blood from all participants, centrifuged at 3500 rpm for 10 min at 4 °C to separate plasma, transferred the supernatant to an EP tube and stored it in a − 80 °C refrigerator, and used enzyme-linked immunosorbent assay (ELISA) to measure inflammatory indicators such as C-reactive protein(CRP), interleukin-1β(IL-1β), interleukin 6(IL-6), and tumor necrosis factor-α(TNF-α). The experimental procedure of ELISA: (1)Thaw the plasma samples naturally at room temperature. (2)Set up blank holes, standard holes, and sample holes. Add 50µL of different concentrations of standard solution to the standard holes, and add 50µL of the test sample to the sample holes. Leave the blank holes empty. (3)Except for the blank holes, add 100µL of detection antibody labeled with horseradish peroxidase (HRP) to each well of the standard and sample holes. Seal the reaction holes with a sealing film and incubate at 37 °C in a constant temperature chamber for 60 min. (4)Discard the liquid, tap dry on absorbent paper, fill each hole with wash buffer (350µL), incubate for 1 min, remove the wash buffer by flicking, tap dry on absorbent paper, repeat this washing step for the plate 5 times. (5)Add 50µL of substrate chromogen A and B to each hole, and incubate at 37 °C in the dark for 15 min. (6)Add 50µL of stop solution to each hole to terminate the reaction. Within 15 min, measure the OD values of each hole at a wavelength of 450 nm. (7)Plot a standard curve with the concentration of the standard as the x-axis and the corresponding OD values as the y-axis. Perform linear regression analysis on the standard curve and calculate the concentration values of each sample according to the curve equation.

### 16 S rRNA gene sequencing and bioinformatics data analysis

Fecal DNA was extracted using a Stool Mini Kit (QIAGEN, Hilden, Germany) according to the manufacturer’s instructions. DNA integrity and purity were measured using 1% agarose gel electrophoresis. To amplify the bacterial 16 S rRNA gene, we used 338 F universal primers (F: ACTCCTACGGGAGGCAGCA) and 806R (R: GGACTACHVGGGTWTCTAAT) to amplify bacterial 16 S rRNA hypervariable regions V3-V4. The Illumina NovaSeq PE250 platform (Personal Biotechnology Co., Ltd., Shanghai, China) was used for PCR amplification and sequencing library preparation. QIIME 2 (version:2019.1) was used to process and analyze 16 S rRNA raw sequencing data, including quality filtering, denoising, sample inference, clustering, chimera identification, and removal. Sequences with 100% similarity were clustered into amplicon sequence variants (ASVs) and the abundance and classification of all ASVs for each specimen were recorded. The Chao1, Faith pd, Goods Coverage, Shannon, Simpson, Pielou, and observed species indices were used to evaluate the alpha diversity index of the microbiome. Among them, the Chao1 and observed species indices represent the richness of the flora, which only considers the number of species and not the abundance of each species in the community. The Goods Coverage index refers to microbial coverage, and the Simpson, Pielou, Shannon, and Faith Pd indices are used to reflect species diversity, including species richness and evenness. Beta diversity analysis was performed using the principal coordinate analysis (PCoA) based on Bray Curtis to determine differences in microbiome composition at the ASVs level between the groups. Linear discriminant analysis effect size (LEfSe) was used to identify the microbiota that most likely explained the differences between the groups.

### Statistical analyses

All analyses were performed using SPSS Statistics Version 23.0. Shapiro-Wilks test and Levene test were used to perform normality test and variance homogeneity test on continuous data such as age, BMI, years of education, HAMD-24 scores and inflammatory factor levels. One-way analysis of variance (ANOVA) and post hoc LSD test were used to detect differences between groups for continuous data such as age, years of education, BMI, and inflammatory factor levels that follow a normal distribution and have homogeneity of variance, with the results expressed in the form of mean ± standard deviation. The Kruskal-Wallis H test and post hoc Nemenyi test were used to detect differences between groups for continuous data such as HAMD-24 scores that does not follow a normal distribution or have homogeneity of variance, with the results expressed in the form of median(quartile range). The χ^2^ test was used to evaluate gender differences between groups. As the abundance of gut microbiota, HAMD scores were not normally distributed, Spearman’s rank correlation analysis was used to determine the correlation between gut microbiota and inflammatory factors, HAMD scores, and anorexia. For all the test results, a *p*-value less than 0.05 was considered statistically significant (two-tailed).

## Results

### Demographic characteristics among the three groups

Table 1 presents the general demographic characteristics of the three groups. There were no significant differences in age, sex, years of education, and BMI among the three groups (*P* > 0.05). The HAMD scores of MDD patients is significantly higher than that of HC (*P* < 0.001), and no significant difference showed in HAMD scores between the MDA0 and MDA1 group (*P* > 0.05).

**Table 1:** General data comparison of the three groups

Variable	HC	MDA0	MDA1	F/χ^2^/H	*P* Value
Gender (M/F)	14/32	5/15	9/17	0.494	0.781^a^
Age (years; means ± SD)	26.000 ± 5.300	26.850 ± 4.704	27.962 ± 5.040	1.232	0.297^b^
BMI (Kg/m^2^; means ± SD)	22.337 ± 3.423	22.162 ± 3.412	22.709 ± 3.282	0.166	0.847^b^
Years of education(means ± SD)	16.413 ± 3.030	15.550 ± 2.089	16.346 ± 2.911	0.697	0.501^b^
HAMD-24(median(QR))	1.000(2.000)	22.000(2.000)	25.500(10.250)	70.917	< 0.001^c^

HC: healthy control group; MDA0: MDD without anorexia; MDA1: MDD with anorexia

a *P* value is χ^2^ test results

b *P* value is the result of ANOVA test among multiple groups

c *P* value is the result of Kruskai-Wallis H test among multiple groups

### Characteristics of inflammatory factors in the three groups

There was no significant difference in IL-1β, IL-6, and TNF-α among the three groups of subjects (*P* > 0.05). CRP was higher in the MDA0 and MDA1 than HC group (*P* < 0.05) and there was no significant difference between the MDA1 and the MDA1 group (*P* > 0.05)(Table 2).

**Table 2:** Comparison of inflammatory factors among three groups of subjects

Variable	HC	MDA0	MDA1	F	*P* Value
CRP(ng/ml; means ± SD)	102.692 ± 20.923	138.510 ± 23.452	135.070 ± 19.915	29.462	< 0.001
IL-1β(pg/ml; means ± SD)	182.120 ± 34.557	184.050 ± 46.294	176.054 ± 37.729	0.300	0.741
IL-6(pg/ml; means ± SD)	122.385 ± 21.010	110.207 ± 25.038	115.951 ± 25.764	2.031	0.137
TNF-α(pg/ml; means ± SD)	490.700 ± 106.104	496.357 ± 00.899	493.552 ± 93.041	0.023	0.977

HC: healthy control; MDA0: MDD without anorexia; MDA1: MDD with anorexia

*P* value is the result of ANOVA test among multiple groups

### Gut microbiota diversity index among the three groups

**Fig. 1:**
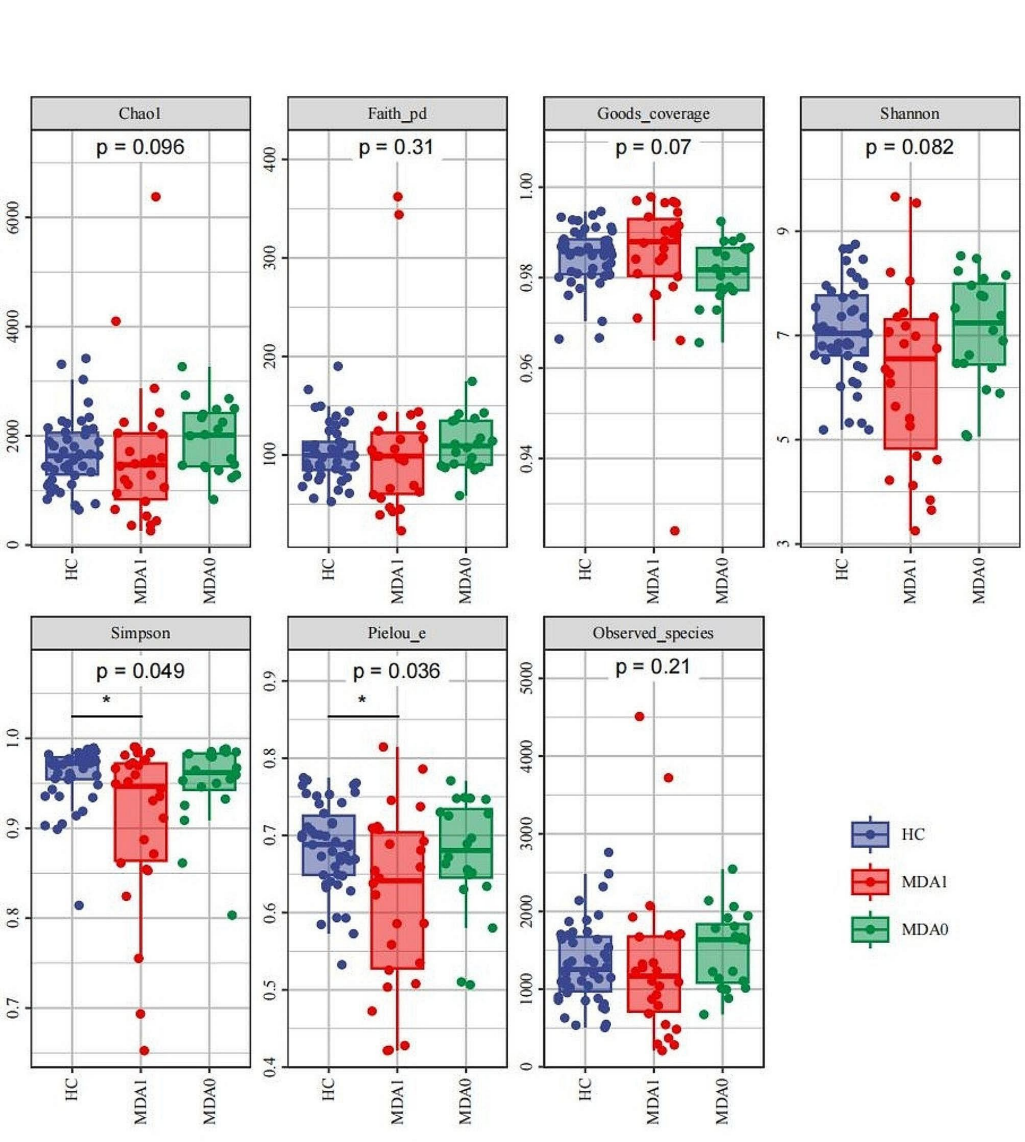
α-Diversity of HC group, MDA1 group and MDA0 group. *P < 0.05

**Fig. 2:**
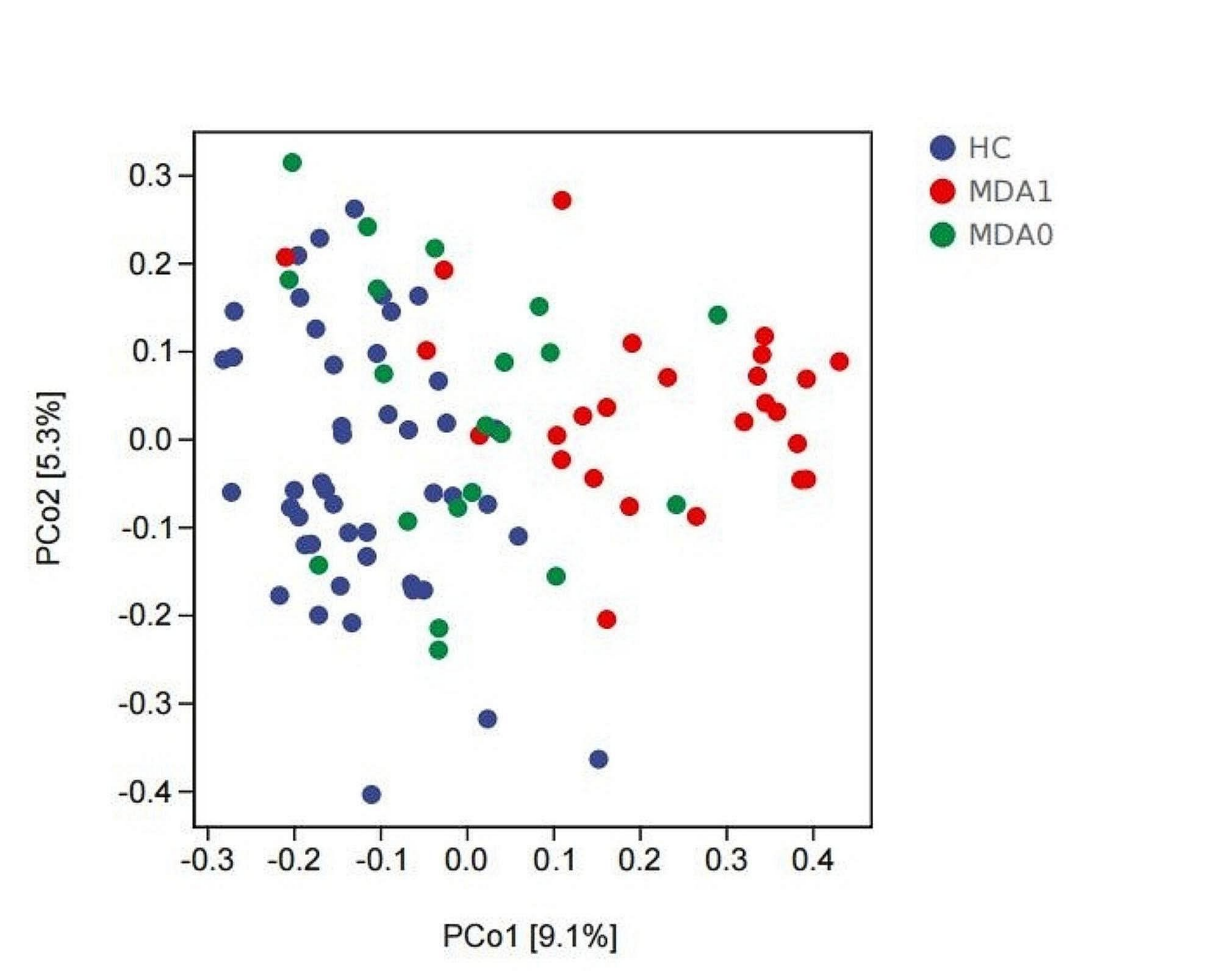
PCoA of beta diversity based on bray_curtis. Blue represents the HC group, red represents the MDA1 group, and green represents the MDA0 group

Alpha diversity analysis of the HC, MDA0, and MDA1 group revealed that the Simpson and Pielou indices of the HC group were higher than those of the MDA1 group (*P* < 0.05), and there were no significant differences in the other indicators among the three groups (*P* > 0.05) (Fig. 1). Using beta diversity as a PCoA based on Bray_Curtis, we found that there were differences in the composition of the microbial communities among the MDA0, MDA1, and HC group (Fig. 2).

### Identification of different species

**Fig. 3:**
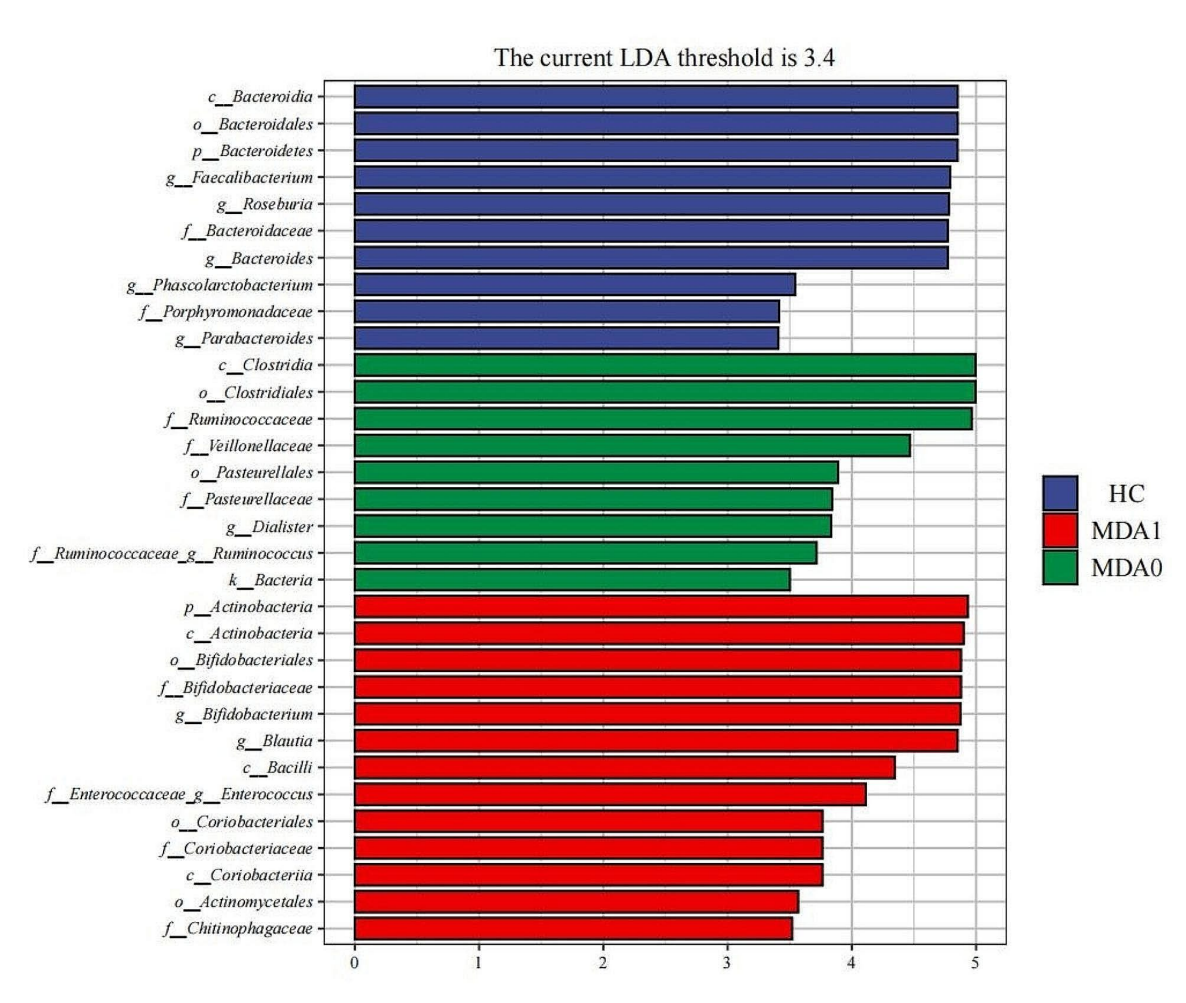
The biomarkers of the HC, MDA1, and MDA0 groups were found by the LEfSe analysis. Blue, red and green represent the enriched populations of the HC, MDA1, and MDO groups, respectively. The p, c, o, f, and g before the microbial name represent the phylum, class, order, family, and genus, respectively. Only taxa with *P* < 0.05 and a LDA score over 3.4 are shown

LEfSe analysis was used to identify microbial differences among the three groups. The results (Fig. 3) showed that 10 microbial communities were enriched in the HC group, 9 in the MDA0 group, and 13 in the MDA1 group. At family level, the HC group showed the highest abundance of Bacteroidaceae. Ruminococcaceae and Bifidobacteriaceae were the most abundant genera in the MDA0 and MDA1 groups, respectively. At genus level, the MDA0 group had higher abundances of Dialister and Ruminococcus; the MDA1 group had higher abundances of Blautia, Enterococcus, and Bifidobacterium; and the HC group had higher abundances of Faecalibacterium, Bacteroides, Roseburia, Phascolarctobacterium, and Parabacteroides.

### Relationships between gut microbiota and inflammatory factors, HAMD scores, and anorexia

**Fig. 4:**
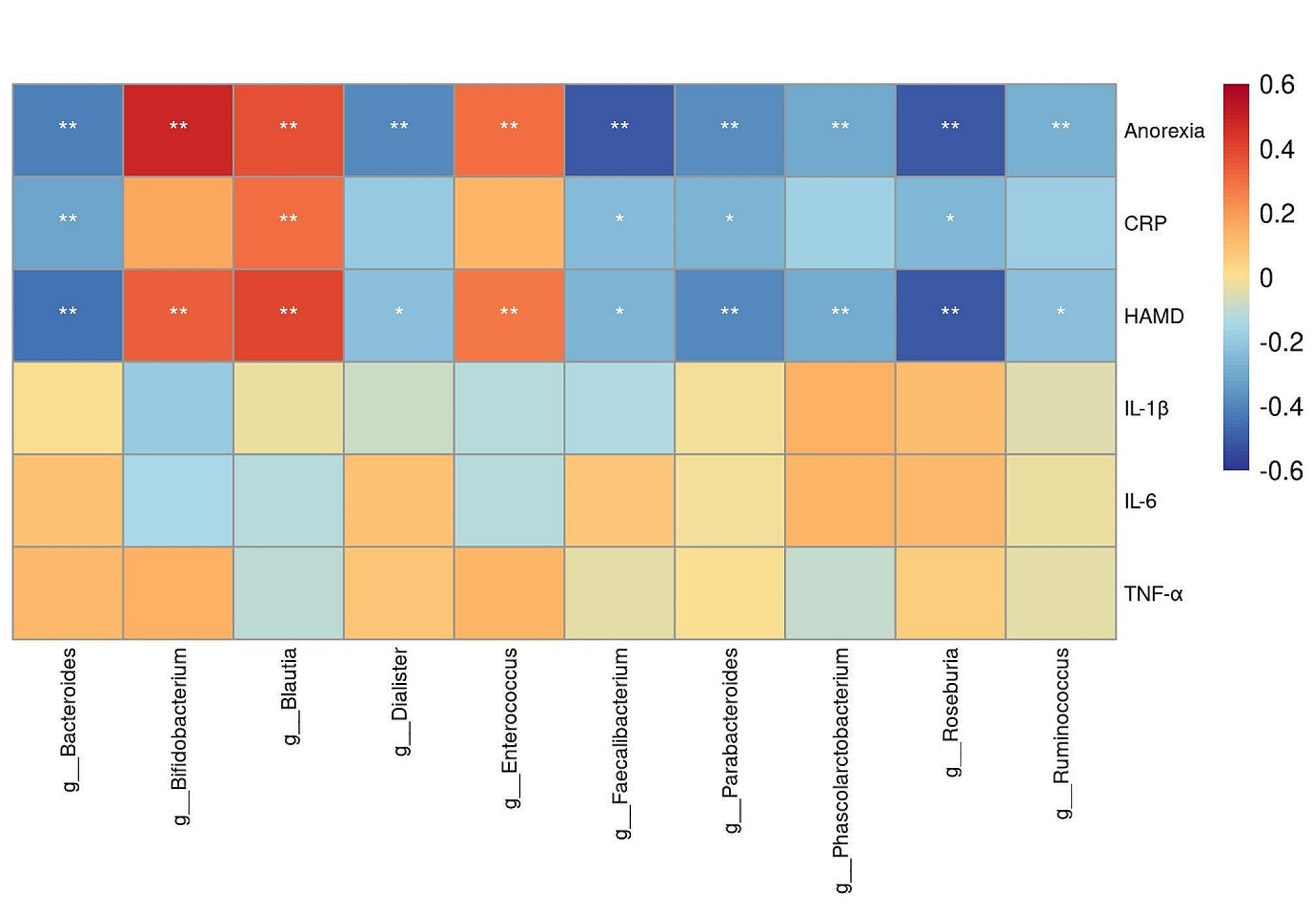
Heatmap of the spearman correlation coefficient matrix between significantly different gut microbiota and inflammatory factors, HAMD scores, and anorexia. Red indicates a positive correlation and blue indicates a negative correlation. *P < 0.05, **P < 0.01

We analyzed the correlation between the representative microbiota and inflammatory factors, HAMD scores, and anorexia among the three groups. The results showed that Blautia was positively correlated with anorexia, HAMD scores, and CRP level, whereas Faecalibacterium, Bacteroides, Roseburia, and Parabacteroides were negatively correlated with anorexia, HAMD scores, and CRP level(Fig. 4). Furthermore, we did not see a correlation between the microbiota and IL-1β, IL-6, and TNF-α.

### Receiver operating characteristic (ROC) curve

**Fig. 5:**
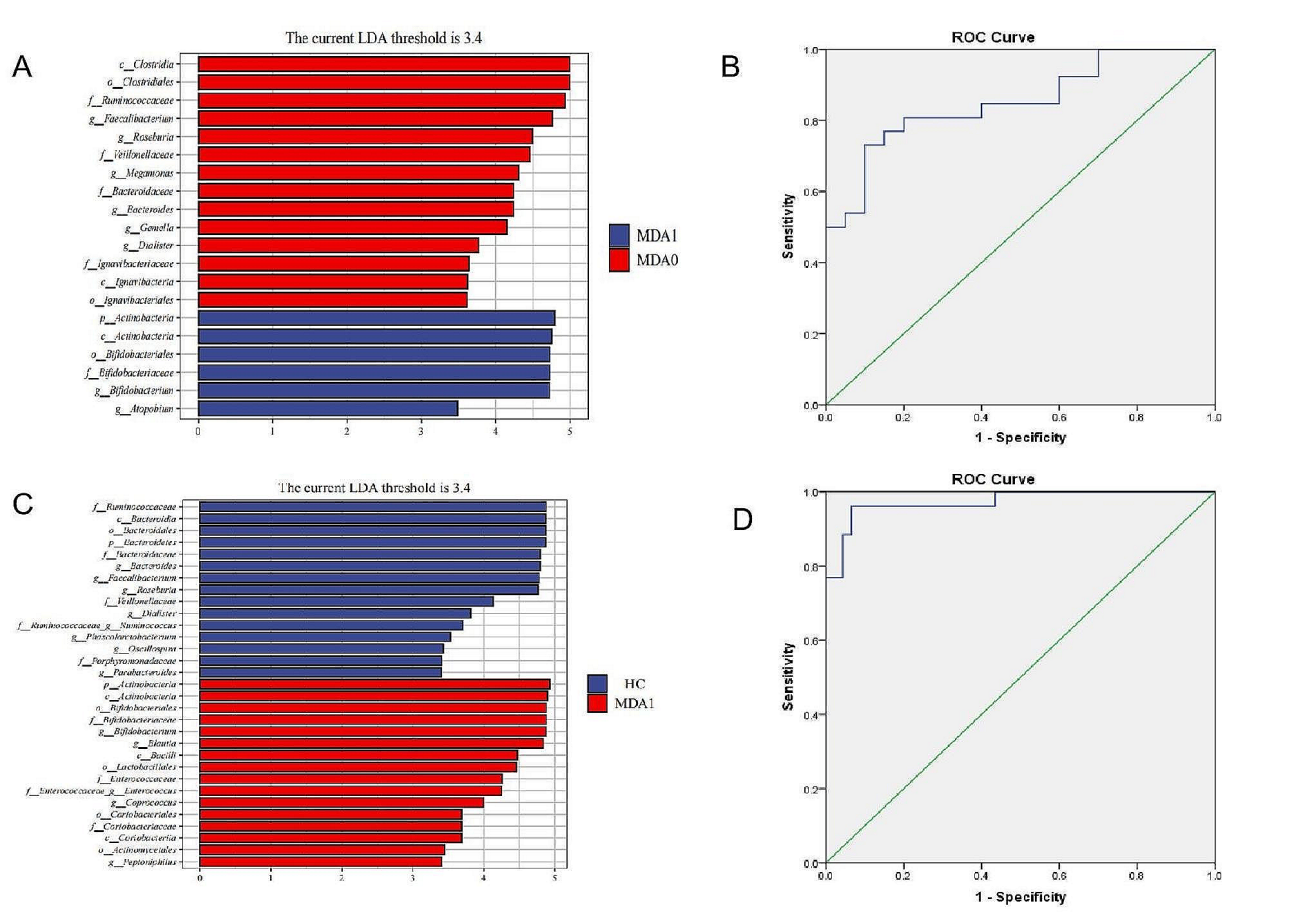
**A, B**: The differential bacterial genera between the MDA1 group and the MDA0 group and their ROC curves as biomarkers to identify whether MDD patients are accompanied with anorexia, with an AUC of 0.85; **C, D**: The differential bacterial genera between the MDA1 and the HC groups and their ROC curves as biomarkers to diagnose MDD patients accompanied with anorexia, with an AUC of 0.97

The representative microbiota of the MDA1 and MDA0 groups were identified using the LEfSe analysis. These were mainly attributed to eight genera at the genus level, two of which were highly expressed in the MDA1 group: Atopobium and Bifidobacterium, and six were highly expressed in the MDA0 group: Dialister, Gemella, Bacteroides, Megamonas, Roseburia, and Faecalibacterium. A ROC curve was drawn using eight bacterial genera as biomarkers to identify whether MDD patients were accompanied with anorexia. The AUC, sensitivity, and specificity were 0.85, 90%, and 73.1%, respectively. The representative microbiota of the MDA1 and HC groups were identified using the LEfSe analysis. They were mainly attributed to 13 genera at the genus level, five of which were highly expressed in the MDA1 group: Peptoniphilus, Coprococcus, Enterococcus, Blautia, and Bifidobacterium; eight highly expressed in the HC group: Parabacteroides, Oscillospira, Phascolarctobacterium, Ruminococcus, Dialister, Roseburia, Faecalibacterium, and Bacteroides. A ROC curve was constructed using 13 bacterial genera as biomarkers for the diagnosis of MDD patients with anorexia. The AUC, sensitivity, and specificity were 0.97, 93.4%, and 96.2%, respectively (Fig. 5).

## Discussion

As mentioned before, MDD and anorexia often occur simultaneously. Therefore, we analyzed the basic clinical conditions, inflammatory factors, and gut microbiota characteristics of MDD patients with anorexia and further analyzed the correlation between different bacterial genera and inflammatory factors, HAMD scores and anorexia. The results showed that MDD patients with anorexia had an altered gut microbiota, and some bacterial genera correlated with inflammatory factors, HAMD scores, and anorexia .

Many studies show that the inflammatory response plays an important role in the occurrence and development of MDD [16, 18]. In addition, there is a certain correlation between depression and serum inflammatory factors such as IL-6, TNF-α, and CRP [23]. Our study found that compared with the HC group, the peripheral blood CRP of MDD patients was elevated, and there were no changes in IL-1β, IL-6, and TNF-α. This is inconsistent with the results of previous related studies and may have been caused by factors such as the sample source of each study, basic inflammation level of the sample, and testing method. CRP is an acute-phase protein that plays an important role in infection, immunity, and inflammation. It is a biomarker of MDD patients. In treatment-resistant MDD patients, the increase in CRP was more obvious [24]. Depression with peripheral blood CRP > 3 mg/L is usually considered inflammation-related [25], and patients with this type of MDD are more likely to show treatment resistance, probably due to inflammatory factors block and destroy the action pathways of antidepressants [24, 25]. We further analyzed the serum CRP level of MDD patients with or without anorexia and found no difference in CRP level between the MDA1 and MDA0 groups. Research has shown that appetite is related to CRP [26], but another study showed that poor appetite is related to CRP in male patients, however, it has nothing to do with CRP in female patients [27]. It may be that relevant research subjects mainly focused on patients with tumors, whereas we were more concerned about MDD patients. Different underlying diseases can result in varying results. In addition, studies have shown that different subtypes of MDD have different inflammatory characteristics [28, 29]. The levels of inflammatory factors in MDD subtypes that are accompanied with anorexia do not change significantly compared with those in MDD patients. In this study, the sample size was small, which may have influenced the results.

Relevant studies have shown that changes in gut microbiota are closely related to MDD. The analysis of α-diversity in this study revealed that the Simpson and Pielou indices of MDD patients with anorexia were lower than those of healthy controls. The other indicators reflecting alpha diversity showed no obvious abnormalities. Therefore, we hypothesized that gut microbiota α-diversity may be reduced in MDD patients compared with healthy controls. At present, the research results on the changes in α-diversity of gut microbiota in depression are inconsistent. Liu et al. [30] found that depression patients showed lower α-diversity comparing with healthy controls. In another study, Jiang’s research showed that depression patients have a higher α-diversity index than healthy people [31]. In addition, some studies have shown no difference in α-diversity between depression patients and healthy controls [32]. Similar to α-diversity, the current research results on β-diversity in depression patients are also inconclusive. Most studies have found differences in β-diversity between depression patients and healthy controls [30, 33], while some studies have shown no difference between depression patients and healthy controls [31]. Our findings show difference in β-diversity between depression patients with anorexia and healthy individuals or depression patients without anorexia, which is not entirely consistent with related findings. This may be caused by factors such as different subtypes of depression, different sequencing methods, and different courses of depression.

Our current study shows a difference in gut microbiota α-diversity between MDD patients with anorexia and HC, but no difference in MDD patients with or without anorexia. However, there were differences in the β-diversity of gut microbiota among the three groups. Relevant animal experiments [34] and clinical studies [35] have shown that anorexia is accompanied by changes in gut microbiota, which is consistent with our findings. Gut microbiota or microbial-derived short-chain fatty acids (SCFAs) mainly affect appetite via central and hormonal signals. For example, the gut microbiota regulates appetite by affecting leptin signaling, insulin signaling, and mediating bile acids, in addition, gut microbes can regulate gastric hunger signals or related neurotransmitters to achieve appetite regulation [36, 37].

Besides, we found that compared to MDD patients, HC individuals have higher abundance of Faecalibacterium, Bacteroides, Roseburia, Phascolarctobacterium and Parabacteroides. There are also related research confirming the higher abundance of these genera in healthy subjects [38]. Faecalibacterium, Bacteroides, Roseburia, Phascolarctobacterium, and Parabacteroides are all beneficial bacteria that contribute to host health [39, 40], and they also have the ability to ferment and produce SCFAs in the human intestinal tract [40, 41]. SCFAs play a crucial role in the interaction of the gut-brain axis. Peripherally, SCFAs promote gut health by maintaining intestinal barrier integrity, stimulating mucus production, and exerting anti-inflammatory effects. In the CNS, SCFAs also play a regulatory role by influencing the morphology and function of glial cells, modulating neurotrophic factor levels, promoting neurogenesis, enhancing serotonin synthesis, and improving neuronal homeostasis and function to impact the development of neuroinflammation [42]. SCFAs also play a crucial role in regulating body metabolism, exerting significant effects on adipose tissue metabolism, lipid oxidation capacity, and insulin secretion [43]. Therefore, the presence of these bacteria in the healthy population plays a crucial role in maintaining human health.

We further analyzed the characteristics of the gut microbiota in MDD patients with anorexia and found that the abundance of Blautia, Enterococcus, and Bifidobacterium in the MDA1 group was higher than that in the HC and MDA0 groups. Joo et al. found that Enterococcus in MDD patients was significantly higher than that in normal people and was a risk factor for depression [44], which is consistent with our results. Blautia and Bifidobacterium are generally considered beneficial bacteria, have certain anti-inflammatory effects, and are found in high amounts in healthy individuals [45, 46]. In addition, some types of Bifidobacteria have been used to relieve depressive symptoms and prevent depression [45], which is inconsistent with our findings. This inconsistency may be because not all Blautia and Bifidobacterium species are probiotics. Choy [47]and Nishino [48] found a higher abundance of Blautia and Bifidobacterium in patients with psoriasis and ulcerative colitis. Chung [49] found that Blautia and Bifidobacterium were more abundant in MDD patients than in healthy individuals, and some studies have shown that Bifidobacterium can induce a TH2-driven immune response [50]. Since inflammation plays an important role in the pathogenesis of depression, Blautia and Bifidobacterium may cause depression by inducing inflammatory responses in the body. However, we found that Dialister and Ruminococcus in MDD patients without anorexia were not associated with any inflammatory factors and negatively correlated with HAMD scores. Therefore, Dialister and Ruminococcus may influence the onset of depression through mechanisms other than inflammation. Some gut microbiota have the ability to synthesize hormones such as serotonin, dopamine, and gamma-aminobutyric acid, which play crucial roles in the brain-gut interaction. Metabolites produced by the gut microbiota can alter the permeability of the intestinal mucosa and the blood-brain barrier, thereby influencing the CNS. Furthermore, the gut microbiota can also modulate the signaling of the vagus nerve to impact the CNS [15]. These mechanisms mediated by gut microbiota also significantly impact the occurrence and development of depression.

A correlation analysis showed that Bifidobacterium, Blautia, and Enterococcus were significantly positively correlated with HAMD scores. Previous studies by our research group have also shown that the abundance of Bifidobacterium and Blautia were positively correlated with HAMD scores [51]. As the comparison of inflammatory factors, IL-1β, IL-6, and TNF-α had no significant differences between the HC, MDA0, and MDA1 group. Besides, there was no correlation between microbiota and IL-1β, IL-6, and TNF-α, therefore, we focus on the correlation between microbiota and CRP. We found that the abundance of Blautia was positively correlated with anorexia, HAMD scores, and CRP level, whereas the abundance of Faecalibacterium, Bacteroides, Roseburia, and Parabacteroides were negatively correlated with anorexia, HAMD scores, and CRP level. This phenomenon has not been reported previously. Faecalibacterium, Bacteroides, Roseburia, and Parabacteroides are bacteria that produce SCFAs such as acetic acid, propionic acid, and butyric acid, all of which have anti-inflammatory and immune-regulatory functions [52–54]. SCFAs, especially butyric and caproic acids, improve intestinal permeability, which can improve the barrier function of the intestinal tract [55]. Studies have shown that SCFAs play an anti-inflammatory role by interfering with the NF-κB pathway, and supplementation with SCFAs can decrease inflammation and relieve depressive symptoms [56]. Therefore, a decrease in SCFAs-producing bacteria in MDD patients may cause depression via an inflammatory response. These inflammatory molecules interfere with appetite and promote anorexia [57]. Inflammation in the hypothalamus has been shown to cause anorexia by upregulating serotonin availability and stimulating its signaling pathways in the hypothalamus [58]. In addition, butyrate can cross the blood-brain barrier and affect appetite and feeding activities of the host by activating the vagus nerve [59]. However, research results on the impact of SCFAs on appetite are not unanimous. Zhang et al. showed that SCFAs can stimulate the colon to secrete satiety hormones and suppress appetite [60]. Whereas, Chen demonstrated that the impact of SCFAs on appetite is not absolute suppression, but may also have an appetite-promoting effect, which is related to the oligosaccharide content in the diet [61]. The impact on appetite may be the result of a combination of factors, and what is relatively clear is that inflammatory factors promote anorexia. Thus, a reduction in anti-inflammatory bacteria in MDD patients may cause anorexia via inflammatory reactions.

Notably, we found that a higher abundance of Blautia in the MDA1 group was positively correlated with anorexia, the HAMD scores, and CRP level. Current research on Blautia are inconsistent. Some studies have shown that Blautia is a probiotic that plays a protective role against various diseases [46]. Another study showed that its abundance is higher in depression patients [49, 51], which is consistent with our findings. Increased Blautia can be seen in some inflammation-related diseases, such as ulcerative colitis [48] and psoriasis [47]. In an obese nonalcoholic steatohepatitis hamster model, Blautia was positively correlated with pro-inflammatory parameters in the lungs and liver [62]. Our study also showed that the abundance of Blautia was positively correlated with serum CRP level, therefore, Blautia may cause depression and anorexia by promoting inflammation. Interestingly, Blautia is also a butyrate-producing bacterium, so it may exert some of the anti-inflammatory effects of SCFAs [46]. The reason for this contradictory result may be that there are different species of Blautia, and the functions of each species are different, or it may be related to the different functions of Blautia in different diseases.

Our study showed that inflammatory factors were associated with MDD and anorexia. Evidence suggested that inflammatory conditions could lead to inflammation-related anorexia and decreased appetite [63], although the specific mechanisms were not yet clearly understood. Some studies indicated that inflammation could impact appetite through various pathways. The lateral hypothalamus(LH) plays a significant role in regulating appetite and hunger sensations, exerting a crucial influence on individual feeding motivation and behavioral patterns [64]. The bed nucleus of the stria terminalis (BNST) has complex cell types and functions, and is connected with brain regions that regulate feeding and energy balance [65]. The oval region of BNST (ovBNST) can suppress appetite by suppressing subregions projected to LH, and inflammatory factors can activate ovBNST, suggesting that inflammation may influence appetite by affecting the microcircuit of BNST [66]. Ghrelin, also known as the hunger hormone, plays a crucial role in regulating appetite, and its level decreases to promote a sensation of fullness [67]. Inflammatory factors can induce anorexia by suppressing the secretion of Ghrelin [68]. Additionally, inflammatory factors can reduce appetite and trigger anorexia by acting on brain receptors [69]. Also, inflammation can affect depression through various pathways. One important mechanism is the overactivation of the tryptophan-kynurenine pathway. Over 90% of tryptophan is metabolized through the kynurenine pathway, with only a small portion being metabolized through the 5-HT pathway. 5-HT, also known as serotonin, is an important neurotransmitter involved in emotional regulation in the brain. Inflammatory factors can overactivate indoleamine 2,3-dioxygenase (a key enzyme in the kynurenine pathway), leading to decreased levels of serotonin in the tryptophan-5-HT pathway and causing depression [70, 71]. Astrocytes and microglia are crucial for maintaining the immune environment of the CNS. Peripheral inflammation can activate astrocytes and microglia, leading to further secretion of inflammatory mediators that impact the function of various neurons. This includes impaired neurotransmitter signal transmission, disruption of neurotransmitter synthesis, reuptake, and release, ultimately causing depression [71, 72]. Furthermore, inflammation can increase oxidative stress, thereby stimulating the production of various pro-inflammatory mediators, leading to the formation of cell pores and substance leakage on the cell membrane, activation of multiple apoptotic factors, and ultimately resulting in neuronal cell death, which can lead to the onset of depression [73]. All of these indicate the association between inflammation and anorexia, as well as MDD. Research suggested that stimulating the cingulate gyrus in its portion below the corpus callosum (SCC) could serve as an effective intervention for treatment-resistant depression, and this region had shown promising results in the treatment of anorexia as well [74]. This implied that anorexia and MDD might be regulated by the same brain region. Xiaoyaosan could ameliorate both depression-like behavior and anorexia behavior induced by chronic unpredictable mild stress (CUMS) in mice [75]. MDD and anorexia can occur as comorbidities, and inflammation may be a common pathogenic mechanism for anorexia and MDD.

We further analyzed the differential bacterial genera between MDD patients with or without anorexia and used them as biomarkers to draw an ROC curve to identify MDD patients with or without anorexia, with an AUC of 0.85. Moreover, we analyzed the differential bacterial genera between MDD patients with anorexia and healthy controls and used them as biomarkers to draw the ROC curve to diagnose MDD patients with anorexia, with an AUC of 0.97. These different bacterial genera may be used in the future to identify whether MDD patients are complicated with anorexia, and to diagnose MDD patients with anorexia.

In conclusion, our study found that depression and anorexia share a common pathogenesis, that is, changes in the gut microbiota may affect depression or appetite through inflammation. The symptoms of anorexia and depression in MDD patients may be physical and psychological manifestations of MDD patients, and MDD patients with anorexia have altered gut microbiota. In the diagnosis and treatment of MDD patients, attention should be paid not only to the psychological but also to the physical performance of the patient. However, genetic, diet, lifestyle, and geographic factors have an impact on the gut microbiota [76]. In future studies on intestinal microorganisms, more rigorous designs are needed to eliminate the impact of related factors on experimental results.

Our study had some limitations. First, we conducted a cross-sectional study, so we can only illustrate the correlation between variables and not their causal relationships. In future research, longitudinal studies may be employed to better understand the causal relationships between variables. Second, the experimenter’s dietary and geographical factors such as dietary habits, lifestyle and environmental exposure may have affected the composition of the intestinal microbiota. Failure to control for these factors could lead to inherent biases in the results, impacting the reliability of the conclusions. In future research, an attempt can be made to eliminate or reduce the bias caused by these factors on the study results by managing the experimenter’s dietary habits and controlling their geographical location. Third, we used 16 S rRNA sequencing technology, which can only detect species at the genus level and cannot detect differences in the microbiota at the species level and may lead to incomplete in understanding the microbial composition. Further research can employ more advanced detection methods to identify the gut microbiota at the species level for a more comprehensive understanding of microbiota composition and a more precise description of microbiota. Fourth, we focused on the relationship between inflammation in the gut microbiota and depression or anorexia, however, hormones, neurotransmitters, and neuromodulation may also be involved in related pathological processes. As this could result in an incomplete understanding of the underlying mechanism and introduce bias, more relevant research is needed to confirm how hormones, neurotransmitters, and neuromodulation affect depression and anorexia. Finally, our sample size was relatively small, which may have affected the accuracy of the experimental results. Expanding the sample size in future study can enhance the stability and reliability of the research.

## Conclusion

This study suggested that MDD patients with anorexia had a distinct gut microbiota compared to healthy individuals, with higher level of CRP. Blautia was more abundant in MDD patients with anorexia and positively correlated with CRP, HAMD scores, and anorexia. The gut microbiota might have influenced MDD and anorexia through the inflammatory factor CRP.

## Data Availability

The data of this study are available from the corresponding author on reasonable request.

## Abbreviations

MDD: Major depressive disorder
HC: Healthy controls
HAMD-24: Hamilton Depression Scale-24
16S rRNA: 16 S ribosomal RNA
CRP: C-reactive protein
ELISA: Enzyme-linked immunosorbent assay
LEfSe: Linear discriminant analysis effect size
ROC: Receiver operating characteristic
AUC: Area under curve
DSM-IV: Diagnostic and Statistical Manual of Mental Disorders, Fourth Edition
BMI: Body mass index
IL-1β: Interleukin-1β
IL-6: Interleukin 6
TNF-α: Tumor necrosis factor-α
ASVs: Amplicon sequence variants
PCoA: Principal coordinate analysis
ANOVA: One-way analysis of variance
SCFAs: Short-chain fatty acids
CNS: Central nervous system
LH: Lateral hypothalamus
CUMS: Chronic unpredictable mild stress
